# Supplementary figures and images for: Advanced glycation end-products reduce lipopolysaccharide uptake by macrophages
Source: PLoS One. 2021 Jan 25;16(1):e0245957. doi: 10.1371/journal.pone.0245957 (PMC7833212; doi:10.1371/journal.pone.0245957)

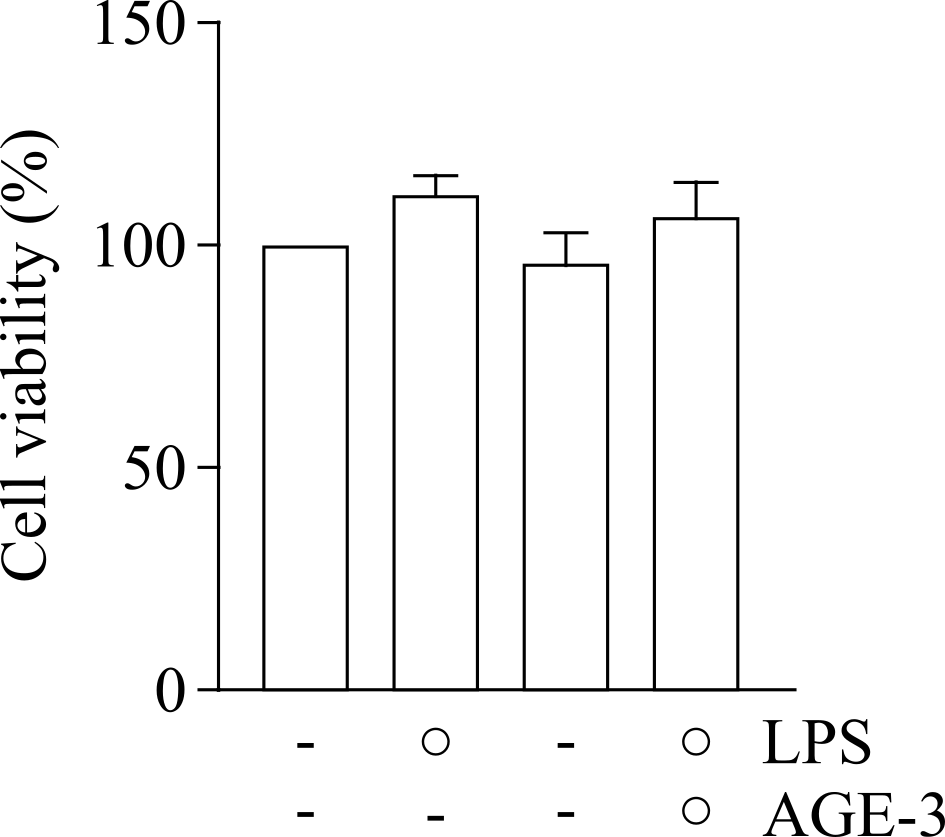

Supplement: S1 Fig — Cells were treated with LPS at 1 μg/mL and/or AGE-3 at 200 μg/mL for 24 h (n = 5 means the number of independent experiments). Data are expressed as means ± SEM and are normalised to the untreated group. (TIF) [file pone.0245957.s001.tif]

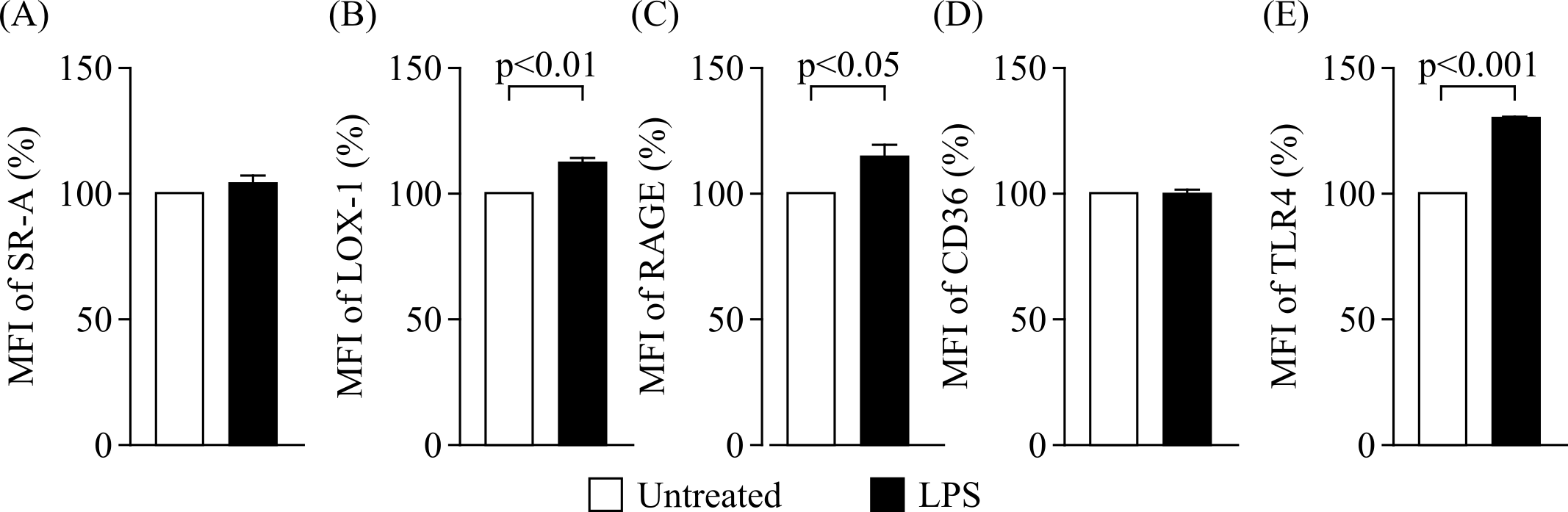

Supplement: S2 Fig — Cells were treated with LPS at 1 μg/mL for 24 h and then the expression of each receptor was determined by flow cytometry. (A) SR-A (n = 4 means the number of independent experiments), (B) LOX-1 (n = 3 means the number of independent experiments, Student’s t-test), (C) RAGE (n = 3 means the number of independent experiments, Student’s t-test), (D) CD36 (n = 3 means the number of independent experiments), and (E) TLR4 (n = 3 means the number of independent experiments, Student’s t-test). Data are expressed as means ± SEM and are normalised to the untreated group. (TIF) [file pone.0245957.s002.tif]

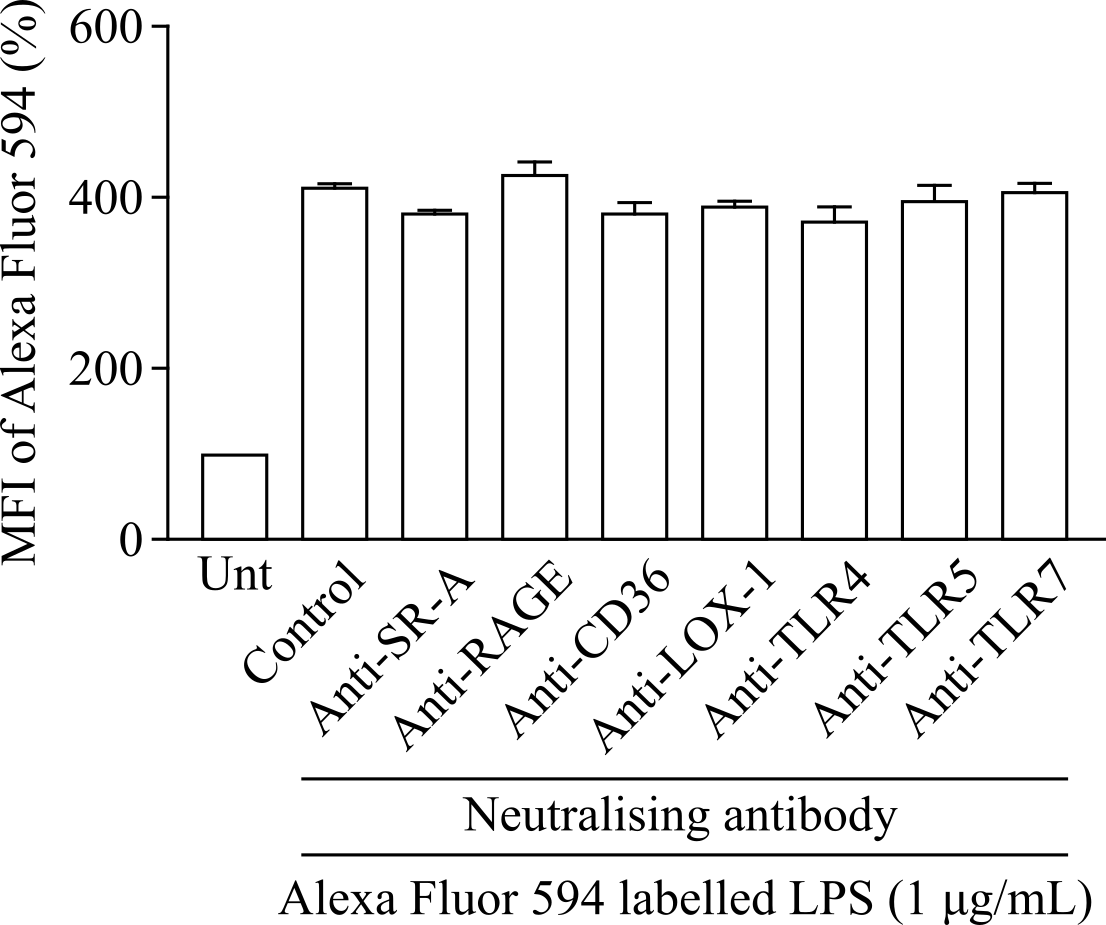

Supplement: S3 Fig — Cells were pre-treated with each neutralising antibody for 1 h before treatment with Alexa Fluor 594-lebelled LPS at 1 μg/mL for 4 h. The MFI was measured by flow cytometry (n = 3 means the number of independent experiments). MFI; mean fluorescence intensity. Unt; untreated. (TIF) [file pone.0245957.s003.tif]

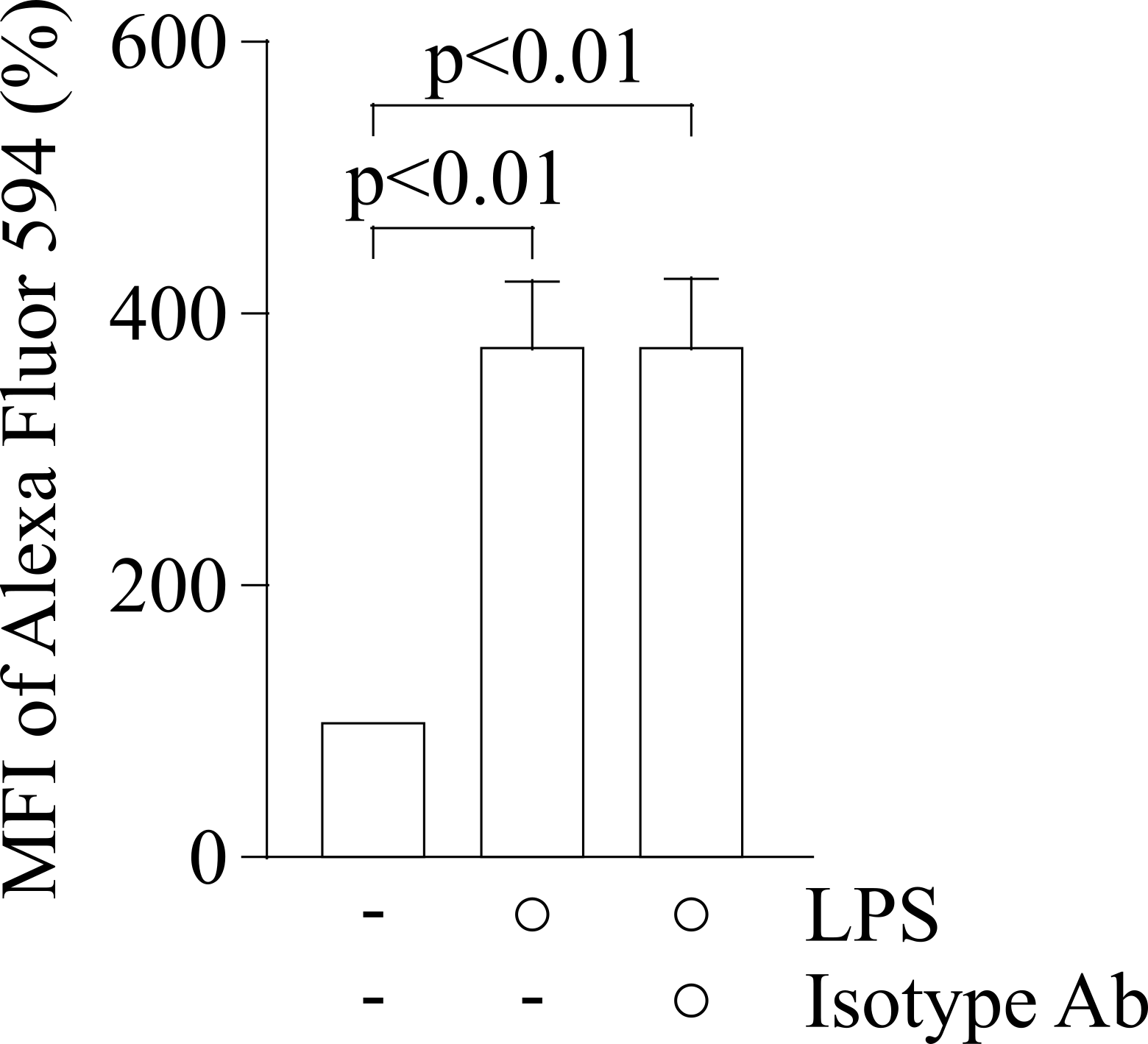

Supplement: S4 Fig — Cells were pre-treated with isotype control antibody for 1 h before treatment with Alexa Fluor 594-lebelled LPS at 1 μg/mL for 4 h. The MFI was measured by flow cytometry (n = 3 means the number of independent experiments). MFI; mean fluorescence intensity. Unt; untreated. (TIF) [file pone.0245957.s004.tif]

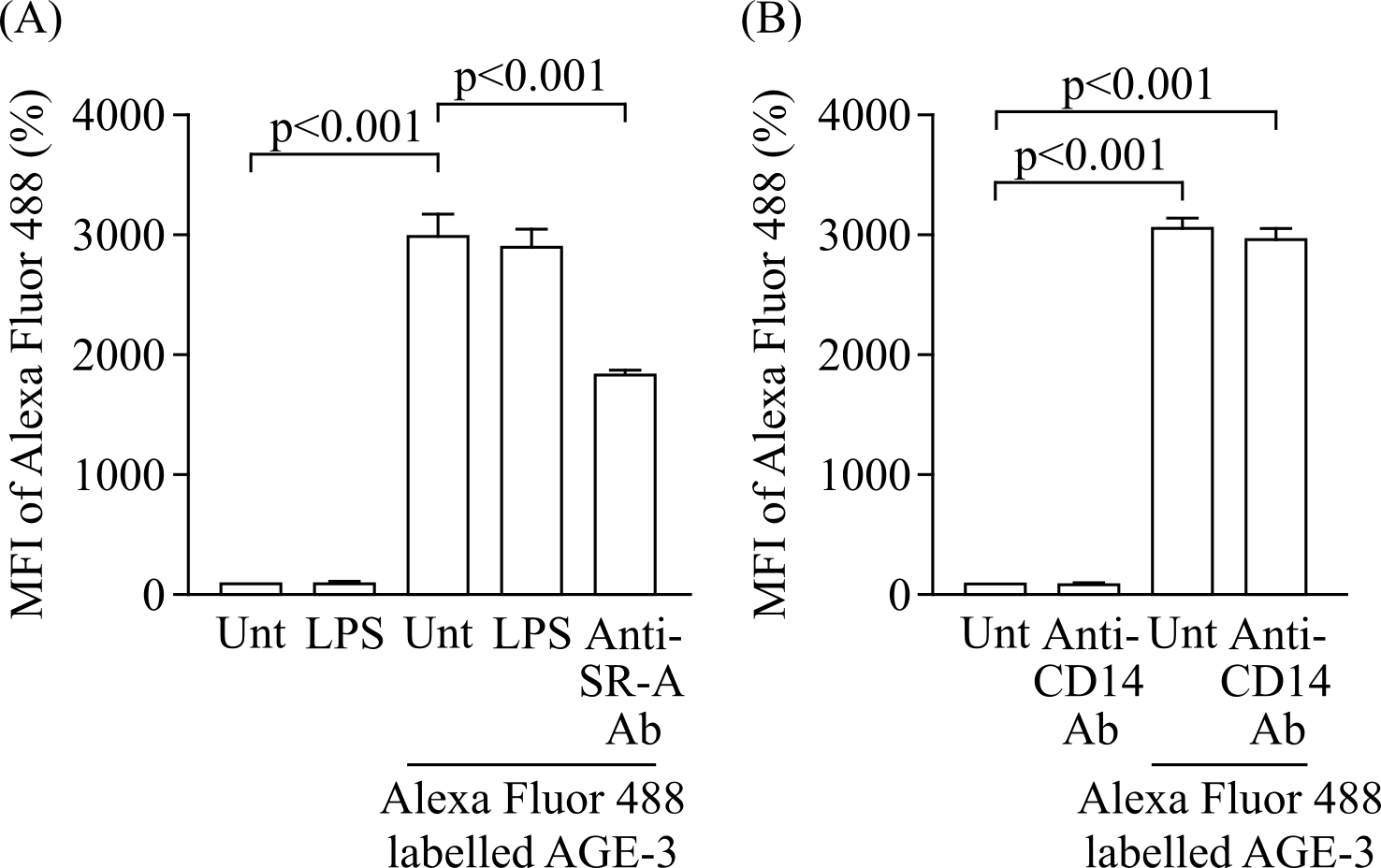

Supplement: S5 Fig — (A) Cells were pre-treated with anti-SR-A antibody for 1 h before treatment with Alexa Fluor 488-labelled AGE-3 at 200 μg/mL. LPS at 1 μg/mL was added concomitant with fluorescent-labelled AGE-3. At 1 h after Alexa Fluor 488-labelled AGE-3 treatment, the MFI was measured by flow cytometry (n = 3 means the number of independent experiments, Turkey’s test). (B) Cells were pre-treated with anti-CD14 antibody for 1 h before treatment with Alexa Fluor 488-labelled AGE-3 at 200 μg/mL. At 1 h after Alexa Fluor 488-labelled AGE-3 treatment, the MFI was measured by flow cytometry (n = 3 means the number of independent experiments, Turkey’s test). MFI relative to the untreated group (medium alone) was arbitrarily defined as 100%. Data are expressed as means ± SEM. MFI; mean fluorescence intensity. Unt; untreated. (TIF) [file pone.0245957.s005.tif]

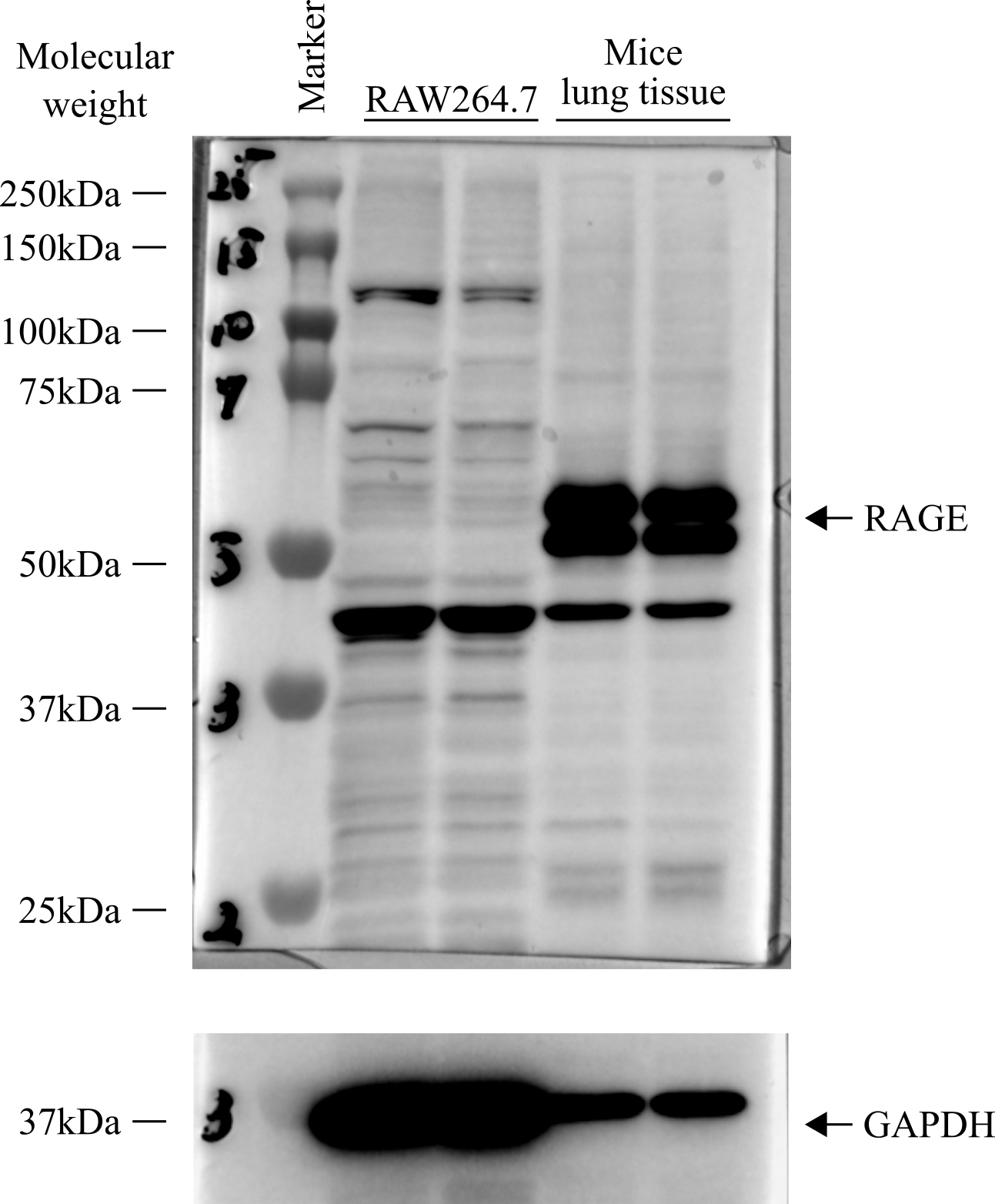

Supplement: S6 Fig — RAGE expression was assessed by using western blot. Representative western blot demonstrating RAGE expression in RAW264.7 cells and mouse lung tissue. The two bands in the vicinity of 50 kDa represent the pre and post glycation type of RAGE protein. GAPDH used as loading control. (TIF) [file pone.0245957.s006.tif]
