# Supplementary material for: Advanced glycation end-products reduce lipopolysaccharide uptake by macrophages
Source: PLoS One. 2021 Jan 25;16(1):e0245957. doi: 10.1371/journal.pone.0245957 (PMC7833212; doi:10.1371/journal.pone.0245957)
Supplement: Attachment — Submitted filename: PLOS one review.docx [file pone.0245957.s007.docx]

This manuscript presents data supporting the conclusion that AGE, working through RAGE, downregulates CD14, thus decreasing LPS uptake and signaling as measured by CX CL10 release. While the results are interesting, they are too preliminary as most of the results are incremental increases or repeats of what is already known. The results are descriptive with no mechanism presented or tested as to how AGE, working through RAGE or SR-A, signal to down-regulate CD14. It is not surprising that down-regulation of CD14 would decrease LPS uptake and it has already been shown that, in monocytes, CD14 is linked to CXCL10 expression. As such, it is this reviewer’s opinion that the data here are too preliminary for publication at this time.

Figure 1 sets up the system, although the conclusion that at 4 hr LPS uptake had “reached maximal level” is misleading as there are no later timepoints to suggest that uptake decreases after that 4 hr (or that it doesn’t continue to rise).

Figure 2. Demonstrates that LPS internalization results in loss of CD14 from the membrane. While internalization of CD14 with LPS may not have been shown before in exactly this way, it is not surprising and thus, represents an incremental increase in our knowledge. Similarly, the ability of CD14 Ab to decrease CXCL10 is derivative of the previous data showing that only CD14^+^ monocytes release CXCL10 in response to LPS (PMID: [19901067](https://www.ncbi.nlm.nih.gov/pubmed/19901067)). How do the authors link the results at 4 hr (CD14, LPS uptake) (Figs 2 A,B) to CXCL10 release in cells treated with LPS and Ab for 24 h (Fig 2C)?

The novelty of this work comes from the impact that AGE have on LPS signaling. AGE reduce LPS uptake although the decrease even at the max does of AGE is modest (although statistically significant) and raises the question of physiological relevance. The representative flow in Fig 3A calls into question whether they is much LPS at all being taken up as there is significant overlap of LPS, LPS+AGE is the untreated and the MFI is so very low. Is the timing right? The longest time used is 4 h; the authors concluded that 4 h was maximal but did not show later timepoints to establish that 4 h represents a plateau of uptake. However, when looking at CXCL10, they incubated the cells with AGE and LPS for 24 h. How do the authors conclude that what happens by 4 h impacts CXCL10 at 24 h in light of the fact that CD14 levels apparently return to normal by 4 h (Fig 2A).

Fig 4. Same comment about timing of CXCL10. All of the results could be predicted from previous published work (that AGE works through RAGE, that AGE decreases LPS uptake and CD14 expression). The novelty is the CXCL10 results but they are done differently than all the other expeirments in the paper so there is not direct link between AGE-CD14-CXCL10. While a cartoon is presented, the critical question remains unanswered: HOW does AGE/RAGE cross-talk with LPS/CD14 to reduce CXCL10 signaling? As such, this body of work is preliminary and needs a more in depth investigation of the signaling network underlying the descriptive results presented.

Statistics: RAW 264.7 is a cell line. Thus, one would expect minimal variation in the results, making n=3 acceptable for the number of experiments run. While a presentation of the variation in 3 points as SEM is published, it is a bit misleading, especially given the very small SEM bars on Fig 3F and Fig 4.

Minor comments:

English in places could be improved

- Figure legend 1 “fluorescence-labelled LPS”, Alex 594-LPS would be better.
- CD14 “is not indispensable for” is a double negative. “CD14 is necessary for” would be clearer.

Fig legend for Fig 3E; lowest concentration of 0.2 as in legend or 2.0 as in bar graph/text?

Figs 3-4 represent the novelty. Many of the controls (ie blocking of other receptors doesn’t alter LPS uptake) could be shown
