# Supplementary material for: Advanced glycation end-products reduce lipopolysaccharide uptake by macrophages
Source: PLoS One. 2021 Jan 25;16(1):e0245957. doi: 10.1371/journal.pone.0245957 (PMC7833212; doi:10.1371/journal.pone.0245957)
Supplement: Attachment — Submitted filename: Response to Reviewers.docx [file pone.0245957.s008.docx]

Response to reviewers:

***1. Please ensure that your manuscript meets PLOS ONE's style requirements, including those for file naming. The PLOS ONE style templates can be found at*** [***https://journals.plos.org/plosone/s/file?id=wjVg/PLOSOne_formatting_sample_main_body.pdf***](https://journals.plos.org/plosone/s/file?id=wjVg/PLOSOne_formatting_sample_main_body.pdf) ***and https://journals.plos.org/plosone/s/file?id=ba62/PLOSOne_formatting_sample_title_authors_affiliations.pdf***

**Response to the requirements:**

We have revised the file name. We have ensured PLOS ONE’s style requirements.

**2. Please ensure you have provided the source, catalog number, and final concentration of all neutralizing antibodies, isotype negative control antibodies, and secondary antibodies.**

**Please also provide the catalog number of the RAW264.7 cells.**

**Response to the requirements:**

We have added the information of cell line. RAW264.7 (EC91062702, DS Pharma Biomedical, Osaka, Japan) (Page 10, Line 4).

***3. We note that you have included the phrase “data not shown” in your manuscript. Unfortunately, this does not meet our data sharing requirements. PLOS does not permit references to inaccessible data. We require that authors provide all relevant data within the paper, Supporting Information files, or in an acceptable, public repository. Please add a citation to support this phrase or upload the data that corresponds with these findings to a stable repository (such as Figshare or Dryad) and provide and URLs, DOIs, or accession numbers that may be used to access these data. Or, if the data are not a core part of the research being presented in your study, we ask that you remove the phrase that refers to these data.***

**Response to the requirements:**

Thank you for valuable suggestions. We have deleted the phrase “data not shown” in our manuscript. The essential data were included in main body of manuscript or supporting information.

***4. In your Data Availability statement, you have not specified where the minimal data set underlying the results described in your manuscript can be found. PLOS defines a study's minimal data set as the underlying data used to reach the conclusions drawn in the manuscript and any additional data required to replicate the reported study findings in their entirety. All PLOS journals require that the minimal data set be made fully available. For more information about our data policy, please see*** [***http://journals.plos.org/plosone/s/data-availability***](http://journals.plos.org/plosone/s/data-availability)***. Upon re-submitting your revised manuscript, please upload your study’s minimal underlying data set as either Supporting Information files or to a stable, public repository and include the relevant URLs, DOIs, or accession numbers within your revised cover letter. For a list of acceptable repositories, please see http://journals.plos.org/plosone/s/data-availability#loc-recommended-repositories. Any potentially identifying patient information must be fully anonymized.***

***Important: If there are ethical or legal restrictions to sharing your data publicly, please explain these restrictions in detail. Please see our guidelines for more information on what we consider unacceptable restrictions to publicly sharing data: http://journals.plos.org/plosone/s/data-availability#loc-unacceptable-data-access-restrictions. Note that it is not acceptable for the authors to be the sole named individuals responsible for ensuring data access. We will update your Data Availability statement to reflect the information you provide in your cover letter.***

**Response to the requirements:**

Thank you for valuable suggestions. The minimal data set were included in main body of manuscript or supporting information.

Response to reviewers:

Reviewer #1

We would like to thank reviewer #1 for the valuable comments and suggestions on our manuscript. We have carefully read the comments and have made the following corrections in the revised version:

***1. Page 6, line 11: What is meant by ‘cross tolerance between AGE and LPS’?***

**Reply to the comment 1:**

We have added the explanation about cross tolerance between AGE and LPS as follows (Page 6, Line 11-12).

Cross tolerance, which priming of macrophage with AGE show decreased responses to LPS, has been established [16], indicating that AGEs is involved in impaired immune responses to microbes.

***2. Do the sample sizes in the figures represent the number of wells/plates? Or unique*** ***batches of cells?***

**Reply to the comment 2:**

In the present study, sample size means independent experiments. We have added the explanation of sample size in each figure legends.

***3. Figure 2 and 3 legend: be sure to define the abbreviation MFI.***

**Reply to the comment 3:**

We have added the abbreviation of MFI in “Material and Methods” and each figure legends as follow. MFI; mean fluorescence intensity.

***4. Figure 3F is interesting. Why do you suppose that AGE-3 inhibits LPS uptake acutely (1 and 2 hours) but not as well at 4 hours?***

**Reply to the comment 4:**

You have raised an important question. The reason why AGE-3 acutely inhibits LPS uptake is unknown. We have added the discussion for acute effect of AGE-3 on LPS uptake and CD14 expression (Page 32, Line 16-Page 33, Line 2).

Furthermore, different effect of AGEs in the presence or absence of LPS may contribute to the acute inhibitory effect of AGEs on LPS uptake and CD14 expression.

***5. Page 25, lines 11-12: Do you mean that the RAGE antagonist prevented the AGE-3 decline in CD14? It is difficult to interpret what you meant by restored downregulation – in my mind that means it helped downregulate CD14.***

**Reply to the comment 5:**

Thank you for your suggestion. Our result indicated that RAGE antagonist prevents AGE-3-induced downregulation of CD14 expression. As you pointed out, we have revised sentence as follows (Page 28 line 4-6).

A RAGE antagonist, FPS-ZM1 at 0.5 and 1 μM, and neutralising antibody against RAGE at 20 μg/mL prevented the AGE-3-induced downregulation of CD14 expression (Figs 4A and 4B).

***6. Page 25, line 16: I did not see a supplemental figure #4.***

**Reply to the comment 6:**

Thank you for pointing out the mistake. We have revised and added the supplementary figure.

S1 Fig. Viability of RAW264.7 cells incubated with LPS and/or AGE-3.

S2 Fig. Effect of LPS on receptor expression on the cell surface of RAW264.7 cells.

S3 Fig. Effect of neutralising antibodies on the LPS uptake in RAW264.7 cells.

S4 Fig. Effect of isotype control for anti-CD14 neutralising antibody on the LPS uptake	in RAW264.7 cells.

S5 Fig. Effect of LPS and neutralising antibodies against SR-A or CD14 on AGE uptake in RAW264.7 cells.

S6 Fig. Protein expression of RAGE in RAW264.7 cells and mouse lung tissue.

***7. Lines 8-9, page 34: It is mentioned that RAGE was not detected using a commercial antibody indicating low expression. Was the antibody validated? Is it possible the antibody was not specific resulting in the low detection?***

**Reply to the comment 7:**

Thank you for comment. We verified specificity and reactivity on commercially available anti-RAGE antibodies (Abcam, #ab3611) by using western blot. The #ab3611 has detected the two bands, which represent pre and post glycation type of RAGE protein, in the vicinity of 50 kDa in mouse lung tissue as positive control. The protein sample prepared from RAW264.7 cells showed weak band in the vicinity of 50 kDa, indicating that RAW264.7 cell shows much lower levels of RAGE compared to lung tissue. Therefore, commercially available anti-RAGE antibodies work to detect RAGE protein. Niven et al reported that RAGE expression of RAW264.7 cells have confirmed at mRNA and protein levels [33]. We suggest that RAGE is a functional receptor for AGEs despite expression are low in RAW264.7 cells.

We have added the materials and methods and the result which indicate much lower protein expression of RAGE in RAW264.7 cells (S6 Fig). We have also revised the results and discussion.

(Results, Page 28, Line 13- Page 29, Line 5)

We verified the protein expression of RAGE in RAW264.7 cells by using western blot. Consistent with previous study [33], the two bands, which represent pre and post glycation type of RAGE protein, in the vicinity of 50 kDa were observed in mouse lung tissue used as positive control (S6 Fig). The protein sample prepared from RAW264.7 cells showed weak band in the vicinity of 50 kDa, indicating that RAGE is a functional receptor for AGEs despite expression are low in RAW264.7 cells. Taken together, these findings suggest that AGE-3 attenuated the response to LPS in RAW264.7 cells, which was partially mediated by the activation of RAGE (Fig 5).

(Discussion, Page 32, Line 10-11)

We detected low protein expression of RAGE in RAW264.7 cells.

Reference

33. Niven J, Hoare J, McGowan D, Devarajan G, Itohara S, Gannagé M, et al. S100B Up-Regulates Macrophage Production of IL1β and CCL22 and Influences Severity of Retinal Inflammation. Lewin AS, editor. PLoS One. 2015;10: e0132688. doi:10.1371/journal.pone.0132688

***8. It is not necessary to refer to figure numbers in the discussion section.***

**Reply to the comment 8:**

As you pointed out, we have deleted figure numbers in the discussion section.

***9. Please add a hypothesis to the introduction and abstract.***

**Reply to the comment 9:**

We appreciate your valuable comment. As you pointed out, we have added a hypothesis related to our study in the introduction and abstract as follows.

(Abstract, Page 2, Line 13-15)

Previously, we reported that AGE-3 was internalised into RAW264.7 cells through scavenger receptor-1 Class A. We hypothesized that AGE uptake interrupt LPS uptake and impair innate immune reaction response to LPS in RAW264.7 cells.

(Introduction, Page 6, Line 14-15)

We hypothesized that AGE uptake interrupt LPS uptake and impair innate immune reaction response to LPS in macrophage.

Reviewer #2

We would like to thank reviewer #2 for the valuable comments and suggestions on our manuscript. We have carefully read the comments and have made the following corrections in the revised version:

***1. This manuscript presents data supporting the conclusion that AGE, working through RAGE, downregulates CD14, thus decreasing LPS uptake and signaling as measured by CX CL10 release. While the results are interesting, they are too preliminary as most of the results are incremental increases or repeats of what is already known. The results are descriptive with no mechanism presented or tested as to how AGE, working through RAGE or SR-A, signal to down-regulate CD14. It is not surprising that down-regulation of CD14 would decrease LPS uptake and it has already been shown that, in monocytes, CD14 is linked to CXCL10 expression. As such, it is this reviewer’s opinion that the data here are too preliminary for publication at this time.***

**Reply to the comment 1:**

In the present study, we found that AGE suppressed LPS uptake and CXCL10 production. AGE-induced suppressive effects on the reaction response to LPS were mediated at least in part by reduction of CD14 on the cell surface. Furthermore, blockage of RAGE restored AGE-induced suppressive effects. We believe that our findings also us to propose an importance of AGE-RAGE signaling on impaired innate immune reaction in patients with diabetes. As you pointed out, it is unclear how AGE-RAGE signaling have negatively effects on LPS-CD14 pathway leading to CXCL10 production. Further studies need to investigate molecular mechanisms underlying the effect of AGE-RAGE signaling on impaired innate immune reaction.

**2. Figure 1 sets up the system, although the conclusion that at 4 hr LPS uptake had “reached maximal level” is misleading as there are no later timepoints to suggest that uptake decreases after that 4 hr (or that it doesn’t continue to rise).**

**Reply to the comment 2:**

Thank you for valuable comment. In the present study, we have investigated the LPS uptake by using flow cytometry. When RAW264.7 cells were incubated with LPS for more than 4 h, the analysis could not be accurately performed, because distribution of cell population was confused. This phenomenon might mean cell damage induced by LPS. Although the cell viability measured by WST-8, which is a water-soluble tetrazolium salt and used for assessing cell metabolic activity, did not alter in RAW264.7 cells treated with LPS for 24 h. It is possibility that the manipulation including detachment of cell from plate or centrifugation during the processing of flow cytometry potentially damage cell. In contrast, assessment of cell viability using WST-8 only add detection reagent and is less likely to damage cells. Therefore, we could not assess LPS uptake when cells are exposed to LPS more than 4 h. As you pointed out, it is unclear whether LPS uptake by RAW264.7 cells reach maximal levels at 4 hr.

We have added the discussion about limitation of our study as follows (Page 35, Line 4-14).

Our study has a number of limitations. The first limitation is that we have investigated the LPS uptake by using flow cytometry until 4 h after exposure to LPS. When RAW264.7 cells were incubated with LPS for more than 4 h, the analysis could not be accurately performed, because distribution of cell population was confused. This phenomenon might mean cell damage induced by LPS. Although the cell viability measured by WST-8, which is a water-soluble tetrazolium salt and used for assessing cell metabolic activity, did not alter in RAW264.7 cells treated with LPS for 24 h. It is possibility that the manipulation including detachment of cell from plate or centrifugation during the processing of flow cytometry potentially damage cell. In contrast, assessment of cell viability using WST-8 only add detection reagent and is less likely to damage cells. Therefore, we could not assess LPS uptake when cells are exposed to LPS more than 4 h.

We have revised the results related to time course of LPS uptake as follows (Page 19, Line 10-11).

The mean florescence intensity (MFI) indicating uptake of fluorescently-labelled LPS at 1 μg/ml increased at 2 h (268%) and 4 h (350%).

**3. Figure 2. Demonstrates that LPS internalization results in loss of CD14 from the membrane. While internalization of CD14 with LPS may not have been shown before in exactly this way, it is not surprising and thus, represents an incremental increase in our knowledge. Similarly, the ability of CD14 Ab to decrease CXCL10 is derivative of the previous data showing that only CD14+ monocytes release CXCL10 in response to LPS (PMID: 19901067).**

**Reply to the comment 3:**

In the present study, we found that AGE-3 suppressed LPS uptake in RAW264.7 cells. To determine the effect of AGE-3 on the cell function in RAW264.7 cells related to LPS uptake, we measured several cytokines, which are reported to be upregulated by internalization of LPS, in cell culture supernatant of RAW264.7 cells. We were able to detect CXCL10. As you pointed out, previous reports have demonstrated that neutralizing antibody against CD14 inhibit TNF-α and IL-6 release from macrophage treated with LPS. Our results relating to Fig1 and Fig2 are positioned as the findings to confirm the effect of LPS uptake in RAW264.7.

***4. How do the authors link the results at 4 hr (CD14, LPS uptake) (Figs 2 A,B) to CXCL10 release in cells treated with LPS and Ab for 24 h (Fig 2C)?***

**Reply to the comment 4:**

As the limitation of our study, we evaluated LPS uptake until 4 h, because cell condition is worse (Please see point “Reply to the comment 2” above). We have added the Fig.2C to demonstrate time course of CXCL10 production after LPS treatment. The remarkably increased CXCL10 production in cell culture supernatant were able to be detected by exposure to LPS for 24 h bot not 4, 8 and 16 h. Previous report has demonstrated that upregulation of CXCL10 mRNA by LPS began to increase and reached maximal level at 4 h in peritoneal macrophage prepared from mouse [26]. And then CXCL10 protein are produced by translation and released into culture medium. Therefore, we suggest that the time lag is occurred between CD14-initiated LPS uptake and CXCL10 production. In the present study, our results possibly suggest that AGE-3-induced suppression of CXCL10 release after exposure to LPS is mediated at least in part by inhibition of LPS uptake via downregulated CD14 expression.

We have added the figure to show the time course of CXCL10 production in culture supernatants after exposure to LPS (Fig.2C). We have also added the text relating to the time lag between changes in CD14 expression and CXCL10 production as follows (Page 22, Line 7-15).

We conformed the remarkably increased CXCL10 production in the culture supernatants were able to be detected by exposure to LPS for 24 h bot not 4, 8 and 16 h (Fig 2C). Previous report have demonstrated that upregulation of CXCL10 mRNA by LPS began to increase and reached maximal level at 4 h in peritoneal macrophage prepared from mouse [26]. And then CXCL10 protein are produced by translation and released into culture medium, indicating that the time lag is occurred between CD14-initiated LPS uptake and CXCL10 production. In experimental condition which is exposed to LPS for 24 h, we found that anti-CD14 antibody at 20 μg/ml also suppressed the increased release of CXCL10 in LPS-treated RAW264.7 cells by 79.5% (Fig 2D).

Reference

26. Tebo JM, Kim HS, Gao J, Armstrong DA, Hamilton TA. Interleukin-10 Suppresses IP-10 Gene Transcription by Inhibiting the Production of Class I Interferon. Blood. 1998;92: 4742–4749. doi:10.1182/blood.V92.12.4742

***5. The novelty of this work comes from the impact that AGE have on LPS signaling. AGE reduce LPS uptake although the decrease even at the max does of AGE is modest (although statistically significant) and raises the question of physiological relevance.***

**Reply to the comment 5:**

You have raised an important question. Although we have not answered an important question, we have added the discussion based on our idea as follows (Page 33, Line 6-11).

Although AGE-3-induced inhibition of LPS uptake was little (24% inhibition), the suppressive effect of AGE-3 on CXCL10 levels was much (62% suppression). These results indicate that the effect of AGE-3 on the innate immune reaction in response to LPS have multiple mechanisms of action including inhibition of LPS uptake. In the present study, we found that AGE-3-induced impaired innate immune reaction in response to LPS is mediated, at least in part, by inhibition of LPS uptake.

***6. The representative flow in Fig 3A calls into question whether they is much LPS at all being taken up as there is significant overlap of LPS, LPS+AGE is the untreated and the MFI is so very low. Is the timing right?***

**Reply to the comment 6:**

We have revised Fig.3A. Data altered logarithmic display to biexponential display. Representative histogram shows fluorescence intensity for Alexa Fluor 594-labeled LPS in RAW264.7 cells treated with LPS and AGE-3 for 4 h. The percent of increased fluorescence intensity for Alexa Fluor 594-labelled LPS is 300~350% when untreated data is 100%. Histogram have overlapped each other, since increment of fluorescence intensity is little. The scale of x axis is logarithmic.

***7. The longest time used is 4 h; the authors concluded that 4 h was maximal but did not show later timepoints to establish that 4 h represents a plateau of uptake. However, when looking at CXCL10, they incubated the cells with AGE and LPS for 24 h. How do the authors conclude that what happens by 4 h impacts CXCL10 at 24 h in light of the fact that CD14 levels apparently return to normal by 4 h (Fig 2A).***

**Reply to the comment 7:**

As you pointed out, it is unclear whether LPS uptake by RAW264.7 cells reach maximal levels at 4 hr. Please see point “Reply to the comment 4” above. It is possible that the spending a lot of time is required for LPS-CD14-initiated CXCL10 production due to transcription and translation.

***8. Fig 4. Same comment about timing of CXCL10. All of the results could be predicted from previous published work (that AGE works through RAGE, that AGE decreases LPS uptake and CD14 expression). The novelty is the CXCL10 results but they are done differently than all the other expeirments in the paper so there is not direct link between AGE-CD14-CXCL10. While a cartoon is presented, the critical question remains unanswered: HOW does AGE/RAGE cross-talk with LPS/CD14 to reduce CXCL10 signaling? As such, this body of work is preliminary and needs a more in depth investigation of the signaling network underlying the descriptive results presented.***

**Reply to the comment 8:**

In the present study, for the first time, we found that AGE-3 suppress LPS uptake and CXCL10 levels in response to LPS. These effects are mediated in part by downregulation of CD14. Our findings suggest that impaired innate immune reaction in diabetic patients is associated with increased AGEs and recovered by blockage of AGE-RAGE signaling. As you pointed out, further studies might be needed to clarify the molecular mechanisms regulating CD14 expression by AGE-RAGE signaling.

Thank you for valuable comment. We have added the discussion about further study as follows (Page 35, Line 15- Page 36, Line 2).

Further studies are needed to confirm the inhibitory effect of AGEs on innate immune reaction response to LPS and its molecular mechanisms using human peripheral blood monocyte cell.

***9. Statistics: RAW 264.7 is a cell line. Thus, one would expect minimal variation in the results, making n=3 acceptable for the number of experiments run. While a presentation of the variation in 3 points as SEM is published, it is a bit misleading, especially given the very small SEM bars on Fig 3F and Fig 4.***

**Reply to the comment 9:**

In the present study, number of samples mean independent experiments. As our results are obtained by using cell line, variation of results is small. We have added data in Fig.3F to increase the certainty of our results (n = 5 independent experiments).

**Minor comments**:

***10. English in places could be improved***

***• Figure legend 1 “fluorescence-labelled LPS”, Alex 594-LPS would be better.***

***• CD14 “is not indispensable for” is a double negative. “CD14 is necessary for” would be clearer.***

**Reply to the comment 10:**

Thank you for your suggestion. As you pointed out, we have revised the figure legend. The word alters “fluorescence” to “Alexa Fluor 594”.

We have revised the sentence as follows (Page 21, Line 10-12).

In contrast to CD14, TLR4 contributes to the activation of signalling responses to LPS, although it is not indispensable for LPS uptake.

***11. Fig legend for Fig 3E; lowest concentration of 0.2 as in legend or 2.0 as in bar graph/text?***

**Reply to the comment 11:**

Thank you for your suggestion. We have revised figure legend (Page 26, line 16).

***12. Figs 3-4 represent the novelty. Many of the controls (ie blocking of other receptors doesn’t alter LPS uptake) could be shown.***

**Reply to the comment 12:**

We have added supplementary figure, which shows the effect of neutralising antibody related with scavenger receptors and TLR (S3 Fig). None of neutralising antibody against SR-A, RAGE, CD36, LOX-1, TLR4, TLR5 and TLR7 has no effect on LPS uptake in RAW264.7 cells.

Reviewer #3

We would like to thank reviewer #3 for the valuable comments and suggestions on our manuscript. We appreciate your quick reply to our additional inquiry. We have carefully read the comments and have made the following corrections in the revised version:

***Reviewer #3: The manuscript by Kitaura et al. presents the interdependent effects of AGE3 (a particular type of advanced glycation end-product), lipopolysaccharide (LPS) and the role of the RAGE receptor for the activation of murine macrophages. The authors conclude that RAGE activation by AGE3 impairs immune response in diabetic patients.***

***The described experiments appear to be done carefully and support to overall conclusion of the manuscript. Weak points that should be addressed are the reliance on FACS data for AGE3 and LPS uptake, as well as for cell surface receptor expression. This could be remediated with additional experiments (see details listed below), including additional controls and alternate methodologies (e.g. qPCR / Western Blots for receptor transcription and expression and confocal microscopy for receptor localization. There are some minor correction necessary in the Material / Methods / Experimental sections (details below).***

***The discussion should more clearly point out the relevance of AGE 3 in diabetic patients and the limitations of translating results from cell based assays using mouse cells to human pathology and therapy.***

***The authors should provide additional detail regarding the following:***

**Materials and Methods:**

***1. Page 8 / line 8: What was the concentration of glycoaldehyde used to form AGE-3. How was excess glycoaldehyde removed at the end of the incubation period?***

**Reply to the comment 1:**

We have added information of regarding concentration as follows (Page 8, line 6-12). Briefly, 50 mg/mL of BSA (Fujifilm Wako, Osaka, Japan) was incubated under sterile conditions with 0.2 M of glycolaldehyde (AGE-3) (Sigma-Aldrich) in 0.2 M phosphate buffer (pH 7.4) at 37°C for 7 days. Excess glycolaldehyde removed from AGE-BSA or BSA solution by dialysis for 2 days at 4°C. Cellulose tube 27/32 (UC27-32-100, SEKISUI CHEMICAL, Osaka, Japan) was filled with AGE-BSA or BSA solution and dialysed in 0.02 M PBS.

***2. AGE 8 line 1: What does the acronym SRL stands for?***

**Reply to the comment 2:**

SRL is company name and not acronym (<https://www.srl-group.co.jp/english/>).

***3. PAGE 8: the list of antibodies used in incomplete. See page 13 for other antibodies used.***

**Reply to the comment 3:**

As you pointed out, all antibody used in the present study have listed in “Regents and antibodies” section (Page 9).

neutralising antibodies against RAGE (20 μg/ml, AF1179), LOX-1 (20 μg/ml, AF1564), SR-A/CD204 (20 μg/ml, AF1797, all R&D Systems, Minneapolis, MN, USA), CD14 (20 μg/ml, 557896, BD Biosciences, Franklin Lakes, NJ, USA), CD36 (20 μg/ml, MA5-14112, Thermo Fisher Scientific, Waltham, MA, USA), TLR4 (20 μg/ml, 117608, BioLegend, San Diego, CA, USA), TLR5 (NBP2-24787, Novus Biologicals, Centennial, CO, USA) and TLR7 (NBP2-27332, Novus Biologicals). Anti-mouse Abs against phycoerythrin (PE)-conjugated SR-A (4 ng, 130-102-328, Miltenyi Biotec, Bergisch Gladbach, Germany), PE-conjugated TLR4 (50 ng, 12-9041-80, Thermo Fisher Scientific), PE-conjugated LOX-1 (0.5 µl, FAB1564P, R&D Systems), allophycocyanin (APC)-conjugated CD14 (50 ng, 123312, BioLegend), APC-conjugated CD36 (25 ng, 102612, BioLegend), or APC-conjugated RAGE (50 ng, LS-C212626, LifeSpan BioSciences, Seattle, WA, USA) were used for FACS analysis. Isotype negative control antibodies IgG2a and IgG2b (eBioscience, San Diego, CA, USA) for FACS analysis and against anti-CD14 antibody (Rat IgG2b, 553986, BD Biosciences, Franklin Lakes, NJ, USA) and anti-RAGE antibody (Goat IgG, sc-2028, Santa Cruz Biotechnology, Dallas, TX, USA) were used.

***4. PAGE 10 line 1: What was the final labeling stoichiometry of the Alexa fluorophore bound to AGE-3? BSA has only one free SH group. Is the SH group still accessible after glycation?***

**Reply to the comment 4:**

The final labeling stoichiometry of the Alexa Fluor fluorophore bound to AGE-3 is unclear. The reaction between AGEs and fluorescence-labelling reagent were performed according to manufacturer's protocol. In the present study, each mol of BSA or AGE-3 incubated with almost 20 mol of labeling reagent. Excess labelling reagent is removed by dialysis. AGEs are formed by the Maillard reaction followed by complex reaction including oxidation, dehydration and condensation. The Maillard reaction is generally occurred between a free amino group of protein (e.g. BSA) and carbonyl group (e.g. glycolaldehyde). The products derived from the Maillard reaction finally become AGEs via process of complex reaction. Therefore, free SH group still accessible after glycation.

***5. PAGE10 line 7/8: What does “the strength of Alexa fluor 488-BSA or AGE 3 per unit dosage were adjusted” mean? That may refer in part to Q4***

**Reply to the comment 5:**

Fluorescence intensity per unit dosage are different between fluorescence-labeling BSA and fluorescence-labeling AGE-3. According to previous report [17], fluorescence intensity was adjusted by addition of non-labeling BSA or AGE-3 respectively.

Reference

17. Miki Y, Dambara H, Tachibana Y, Hirano K, Konishi M, Beppu M. Macrophage recognition of toxic advanced glycosylation end products through the macrophage surface-receptor nucleolin. Biol Pharm Bull. 2014;37: 588–596. doi:10.1248/bpb.b13-00818

***6. PAGE 11 line 1: Please identify and list all neutralizing antibodies in “Materials”***

**Reply to the comment 6:**

As you pointed out, all neutralising antibody used in the present study have listed in “Regents and antibodies” section. (Please see point “Reply to the comment 3” above).

***7. PAGE 12 line1: How were cells detached (mechanically or chemically) for FACS analysis.***

**Reply to the comment 7:**

In the present study, cells were mechanically detached by pipetting. We have revised “Materials and Methods” (Page 12, Line 3-4 and Page 13, Line 12).

***8. PAGE 12 line 7 LS-C212626 and RAGE Ab appears to be a polyclonal rabbit anit0human RAGE Ab. How do the authors know that (1) the Ab recognizes mouse RAGE (2) is specific for RAGE (3) shows no cross-reactivity with other cellular proteins. This is particular concerning given that RAGE is apparently expressed at so low levels that it cannot be detected by Western blot.***

**Reply to the comment 8:**

You have raised an important question. In anti-RAGE antibody (LS-C212626) datasheet prepared by LSBio, reactivity to strain is human and mouse. However, we could not confirm the selectivity of this antibody, because we are not able to obtain positive control sample for FACS analysis. We have conformed RAGE expression in RAW264.7 cells by using western blot. Since LS-C212626 conjugate fluorescence substance (Allophycocyanin, APC), we used another anti-RAGE antibody (abcam, #ab3611). The #ab3611 has detected the two bands, which represent pre and post glycation type of RAGE protein, in the vicinity of 50 kDa in mouse lung tissue as positive control. The protein sample prepared from RAW264.7 cells showed weak band in the vicinity of 50 kDa, indicating that RAW264.7 cell shows much lower levels of RAGE compared to lung tissue. Niven et al reported that RAGE expression of RAW264.7 cells have confirmed at mRNA and protein levels [33]. We suggest that RAGE is a functional receptor for AGEs despite expression are low in RAW264.7 cells.

We have added the materials and methods and the result which indicate much lower protein expression of RAGE in RAW264.7 cells (S6 Fig). We have also revised the results and discussion.

(Results, Page 28, Line 13- Page 29, Line 5)

We verified the protein expression of RAGE in RAW264.7 cells by using western blot. Consistent with previous study [33], the two bands, which represent pre and post glycation type of RAGE protein, in the vicinity of 50 kDa were observed in mouse lung tissue used as positive control (S6 Fig). The protein sample prepared from RAW264.7 cells showed weak band in the vicinity of 50 kDa, indicating that RAGE is a functional receptor for AGEs despite expression are low in RAW264.7 cells. Taken together, these findings suggest that AGE-3 attenuated the response to LPS in RAW264.7 cells, which was partially mediated by the activation of RAGE (Fig 5).

(Discussion, Page 32, Line 10-11)

We detected low protein expression of RAGE in RAW264.7 cells.

Reference

33. Niven J, Hoare J, McGowan D, Devarajan G, Itohara S, Gannagé M, et al. S100B Up-Regulates Macrophage Production of IL1β and CCL22 and Influences Severity of Retinal Inflammation. Lewin AS, editor. PLoS One. 2015;10: e0132688. doi:10.1371/journal.pone.0132688

**Statistical Analysis**

***9. The FACS data a generally scaled to a control set to 100% and values for treatments are reported % relative to controls. That means the controls have by default no error or deviation (all 100%). Doing data analysis this way can really cover up large differences between biological independent repeats. It is also not clear if any independent repeats were done. If no independent repeats were done, then scaling to 100% might be okay because then all replicates are just technical replicates. The authors should clarify this point. They should also justify the use of specific post-hoc test, under consideration that controls (defaulted to 100%) have no error.***

**Reply to the comment 9:**

You have raised an important question. In the present study, sample size means independent experiments. In FACS experiment, we obtained data under experimental condition which MFI of untreated sample between each experiment become equal level by adjustment of laser power. There is a variation contributing to cytometer performance including laser power. We have checked cytometer performance every experiment. Therefore, there is few differences contributing to biological independent repeats and we have evaluated the relative effect of treatment.

We agree that the results including data, which defaulted to 100% and have no error, should be analyzed by appropriate statistical test. A randomized block ANOVA is superior to the one-way ANOVA with correlated and uncorrelated data [a]. However, a one-way ANOVA followed by Dunnett’s post hoc test for multiple comparisons is actually used to analysis of the result expressed as percentage of control [b]. It seems that there is no clearly established statistical analysis for data expressed percent of control.

Reference

a. Lew M. Good statistical practice in pharmacology. Problem 2. Br J Pharmacol. 2007;152: 299–303. doi:10.1038/sj.bjp.0707372

b. MD K, KL C, FX L, A D, K T, BM U, et al. Decoy exosomes provide protection against bacterial toxins. Nature. 2020;579. doi:10.1038/S41586-020-2066-6

**Results:**

**Q10: Figure 1 (A) Explain why the untreated cells show two distinct populations, i.e. two FACS peaks.**

**Reply to the comment 10:**

Thank you for pointing out. We have showed the representative histogram plot by logarithmic display. But logarithmic display is not able to represent cell population showing weak fluorescence intensity and result in representation of two peaks as our figure. We have revised Fig.1A and Fig.3A. Data altered logarithmic display to biexponential display. The biexponential display is able to represent cell population showing weak fluorescence intensity and have resulted in representation of one peak. The label of x axis is logarithmic.

***11. Figure 1 (A): Please label both axes of the FACS figure, in particular the fluorescence intensity. That will allow the reader to judge the magnitude of MFI changes shown in the other panels of figure 1.***

**Reply to the comment 11:**

We have added label of both axes in FACS figure (Fig.1A).

***12. Again FACS data are shown as scaled to 100% controls. That makes it impossible to actually judge the sale of treatment related changes. This in turn makes it hard to imply that statistical significance could have biological relevance. While difference s between treatment groups appear to be of up to more than three-fold change, providing actual quantitative data would strengthen the data analysis.***

**Reply to the comment 12:**

In FACS experiment, we obtained data under experimental condition which MFI of untreated sample between each experiment become equal level by adjustment of laser power. There is a variation contributing to cytometer performance including laser power. We have checked cytometer performance every experiment. Therefore, there is few differences contributing to biological independent repeats and we have evaluated the relative effect of treatment. Please see point “Reply to the comment 9” above.

***13. Data interpretation in the written results section is mostly qualitative in nature. It strengthen the paper if the authors would discuss data on a quantitative level.***

**Reply to the comment 13:**

We agree with you. As you pointed out, we have revised the results to reflect the change qualitative to quantitative level in discussing data as follows.

(Page 19, Line 10-11)

The mean florescence intensity (MFI) indicating uptake of fluorescently-labelled LPS at 1 μg/ml increased at 2 h by 268% and 4 h by 350%.

(Page 20, Line 1-3)

In the present study, we found that LPS uptake by RAW264.7 cells was decreased by clathrin-mediated endocytosis inhibitors, sucrose at 0.45 M by 69.6% and chlorpromazine at 20 μM by 57.8% (Fig 1E).

(Page 22, Line 3-4)

Consistent with previous reports, we confirmed that pretreatment with anti-CD14 antibody at 20 μg/ml reduced the LPS uptake by 57.1% (Fig 2B).

(Page 22, Line 13-15)

In experimental condition which is exposed to LPS for 24 h, we found that anti-CD14 antibody at 20 μg/ml also suppressed the increased release of CXCL10 in LPS-treated RAW264.7 cells by 79.5% (Fig 2D).

(Page 25, Line 12-13)

Moreover, AGE-3 at 200 μg/ml also reduced CXCL10 production in the presence of LPS at 1 μg/ml by 62% (Fig 3G).

***14. Figure 3(A) same as Q11, figures need axes labels.***

**Reply to the comment 14:**

We have added label of both axes in FACS figure (Fig.3A).

***15. PAGE 22 line 15: Please provide a more detailed description of how the AGE3 IC50 for LPS uptake was calculated. The four values extractable from figure 3(B) are not sufficient to make such a calculation. Especially since there are not data that justify the statment “ we assumed that the effects …were maximal at 200 ug/ml”. If no additional data are available, the IC50 statement should be removed.***

**Reply to the comment 15:**

As you pointed put, our date is insufficient to calculate IC50. RAW264.7 cells were aggregated by exposure to AGE-3 more than 200 μg/mL, indicating that the examination of dose effect more than 200 μg/mL have difficulty. We agree with your assessment. We have delated IC50.

***16. Figure 3(F): AGE 3 up to 20 ug/ml has no effect on CD14 cell surface level in the presence of LPS (Figure 3F is basically similar to figure 2A). 2 or 20 ug/ml AGE 3 also has no effect on CD14 expression (Figure 3E). IN light of this the statement on PAGE 23 line 9-11 seems poorly supported. In stead, down regulation of CXCL10 could be attributed to AGE3 alone and be independent of CD14.*** ***The following control experiment should be done: measure the effect of AGE 3 on CXCL10 (that would be an additional entry in Figure 4C)***

**Reply to the comment 16:**

You have raised an important question. While AGE-3 at 20 μg/mL significantly inhibited LPS uptake, no significant change in CD14 expression in the absence and presence of LPS was observed in AGE-3 at 20 μg/mL. We believe that AGE-3-induced downregulation of CD14 expression partially contributed to the reduction of LPS uptake and CXCL10 production in response to LPS. As you pointed out, our results also indicated that AGE-3 suppresses LPS uptake and CXCL10 production mediated by CD14 independent mechanisms. We appreciate your valuable comment. We have revised the text (Page 25, Line 13-Page 26, Line 1-2) as follows.

These results suggest that the AGE-3-induced downregulation of CD14 expression partially contributed to the reduction of LPS uptake and CXCL10 production in response to LPS. Furthermore, low dose of AGE-3 inhibited LPS uptake without reduction of CD14 expression in the absence and presence of LPS, indicating that AGE-3 suppresses LPS uptake and CXCL10 production mediated by both CD14 dependent and independent mechanisms.

We have shown the effect of AGE-3 alone on the CXCL10 production in Fig.3G. AGE-3 alone has no effect on CXCL10 production. We believe that the effect of AGE-3 alone on the CXCL10 production is shown in Fig.3G but not Fig.4C, because Fig.3 have referred to the effect of AGE-3. On the other hand, Fig.4 have referred to the involvement of RAGE on the inhibitory effect of AGE-3.

***17. PAGE 25 line 11/12: what is known about the target specificity / off-target effects of FPS-ZM1. Small molecule drugs are notorious for off-target effects. To confirm RAGE involvement additional experiments with monoclonal anti-RAGE antibodies should be done. In essence, the data shown in figures 4A, B and C should include an neutralizing anti-RAGE mAb, including a dose response curve (like 4A).***

**Reply to the comment 17:**

As you pointed out, it is possibility that FPS-ZM1 may inhibit effect of AGE via non-target effect including independent of RAGE inhibition. We have investigated the effect of anti-RAGE neutralising antibody on the AGE-3-induced reduction of LPS uptake, CD14 expression and CXCL10 production. We used polyclonal antibody but not monoclonal antibody, because we have not obtained reliable monoclonal antibody. Anti-RAGE neutralising antibody at 20 μg/mL inhibit the AGE-3-induced reduction of LPS uptake, CD14 expression and CXCL10 production. Control antibody for neutralising antibody has no effect on the CXCL10 production. In addition to results of FPS-ZM1, AGE-3 attenuated the response to LPS in RAW264.7 cells, which was partially mediated by the activation of RAGE. We could not investigate the dose-dependent effect of anti-RAGE neutralising antibody. We agree that it is important to confirm the dose-dependent effect. However, it is difficult to assess the dose-dependent effect of anti-RAGE neutralising antibody, since anti-RAGE neutralising antibody have only a small inhibitory effect.

We have added the results of anti-RAGE antibody (Figs.4B, 4D and 4F) as follows (Page 28, Line 3-13).

In this study, we examined which receptor contributed to the inhibitory effect of AGE-3 on the response to LPS in RAW264.7 cells. A RAGE antagonist, FPS-ZM1 at 0.5 and 1 μM, and neutralising antibody against RAGE at 20 μg/mL prevented the AGE-3-induced downregulation of CD14 expression (Figs 4A and 4B). There was no significant effect of LPS-RS, a TLR4 antagonist, at 10 μg/mL or a neutralising antibody against SR-A (CD204) at 20 μg/mL on CD14 expression and LPS uptake in AGE-3-treated RAW264.7 cells. However, anti-SR-A antibody suppressed the uptake of AGE-3 in RAW264.7 cells (S5 Fig) indicating that the concentration of antibody used was sufficient to suppress effects mediated by AGE-3. Consistent with this, the AGE-3-induced reduction of LPS uptake and CXCL10 release were significantly inhibited by treatment with FPS-ZM1 and anti-RAGE neutralising antibody (Figs 4C-4F).

***18. PAGE 28 line 9/10: What are the absolute RAGE expression levels on RAW624.7 cells? AS mentioned, the anti-RAGE Ab used for FACS seems to be inappropriate (polyclonal, anti-human RAGE). The authors should employ additional confirmatory experiments to show RAGE expression on RAW cells. Why would a Western blot not work when RAGE is there is significant quantities? If WB does not work, show IF micrographs and do an ELISA. FACS as the only method is not convincing.***

**Reply to the comment 18:**

You have raised an important question. Please see point “Reply to the comment 8” above.

***19. Discussion: How do the results obtained from the cultured mouse cells translate to humans with diabetes? Discuss the limitations.***

**Reply to the comment 19:**

We appreciate your valuable comment. We agree your suggestion and have added the discussion as follows (Page 35, Line 15-16 and Page 36, Line 1-2).

The second limitation is that our findings are obtained by a single murine cell line. Further studies are needed to confirm the inhibitory effect of AGEs on innate immune reaction response to LPS and its molecular mechanisms using human peripheral blood monocyte cell.

***20. Discussion: What is the AGE3 (not just “AGE” in general) level in diabetic vs. healthy patients? How does that compare to the 200 ug/ml used in most experiments?***

**Reply to the comment 20:**

You have raised an important question. However, AGE-3 (glycolaldehyde derived AGE) levels in human is unclear. Measurement of AGE-3 have not been established. Glycolaldehyde complexly modify protein and several type of glycation products is formed. The serum AGEs levels in patients with diabetes is almost 80 μg/mL. Therefore, it seems that AGE-3 levels are lower than 80 μg/mL. AGEs are gradually accumulated in tissue, raising the possibility that macrophage within the local microenvironment are exposed to high AGEs levels.

We have added the discussion about AGE-3 levels as follows (Page 31, Line14-17 and Page 32, Line 1-4).

In the present study, we used AGE-3 at 200 μg/mL to examine the inhibitory effect of AGE-3 on the reaction response to LPS. We have no information about the AGE-3 levels in patients with diabetes. Measurement of AGE-3 have not been established. Serum AGEs levels are elevated 2~8-fold (almost 20~80 μg/mL) in patients with diabetes compared with healthy individuals [12,37]. Therefore, it seems that AGE-3 levels are lower than 80 μg/mL. AGEs are gradually accumulated in tissue, raising the possibility that macrophage within the local microenvironment are exposed to high AGEs levels in patients with diabetes.

***21. Figure 5: The figure could suggest that AGE3/RAGE down-regulate CD14 on the genetic level (CD14 expression down regulation). Please clarify. To me, the figure suggests a down-regulation of CD14 on the expression level (genetic down regulation due to reduced transcription / translation). What I think the figure intends to show is the reduction of CD14 levels on the cell surface, possibly due to internalization. The two postulates are not the same. The authors should make very clear what they propose. The data presented (FACS) do not provide evidence for a genetic down-regulation of CD14.***

**Reply to the comment 21:**

Thank you for valuable comment. We appreciate your quick reply to our additional inquiry. We have revised the Fig.5. As you pointed out, AGE-3 suppress RAGE expression on the cell surface but not genetical levels.
