# Supplementary material for: Advanced glycation end-products reduce lipopolysaccharide uptake by macrophages
Source: PLoS One. 2021 Jan 25;16(1):e0245957. doi: 10.1371/journal.pone.0245957 (PMC7833212; doi:10.1371/journal.pone.0245957)
Supplement: Attachment — Submitted filename: Response to Reviewers.docx [file pone.0245957.s009.docx]

Response to reviewers:

Reviewer #2

We would like to express our deepest gratitude reviewer #2 for the valuable comments and suggestions on our manuscript. It was really helpful for us and all suggestions allowed us to improve the quality of our manuscript.

***1. The authors addressed many of the reviewer’s comments although a better discussion of the findings and thoughts on mechanism by which AGE/RAGE impact CD14 and CXCL10 would considerably strengthen the manuscript. Specifically, the validation of the anti-RAGE neutralizing Ab. That the Ab recognizes two large RAGe bands in a positive control is good. However, there are no specific bands corresponding to RAGE in the RAW cells. With this Ab, there are many bands and the most prominent bands are not at 50 kDa. That, coupled with a lack of documentation that the Ab actually binds to anything on the cells (can the receptor be detected by flow?), leaves only the FPS-ZM1 data to implicate RAGE. As FPS-ZM1 is a pharmacological approach, the data is suggestive (could it be blocking something other receptor?, binding and stabilizing CD14 at the cell surface?) but not definitive.***

**Reply to the comment 1:**

Anti-RAGE antibody is able to detect RAGE protein, because the results from western blot analysis showed clearly positive bands in tissue lysate of mouse lung. However, as you pointed out, non-related bands with RAGE were observed in cell lysate of RAW264.7 cells. It is possible that anti-RAGE antibody exhibits inhibitory effect on AGEs though RAGE-non-dependent target. FPS-ZM1 and anti-RAGE antibody partially inhibit AGE-induced downregulation of CXCL10 release from LPS-treated cells, indicating that other target molecules are involved in AGEs effect. High concentration of FPS-ZM1 at 10 μM has inhibitory effect on androgen receptor, benzodiazepine receptor, kappa opioid receptor and melatonin receptor. However, none of these receptors bind to AGEs. In addition, our results showed that FPS-ZM1 alone did not affect LPS-induced CXCL10 release (Fig. 4E). Although our results remain some limitation, we considered that FPS-ZM1 and anti-RAGE antibody inhibit AGEs though blockage of RAGE in the main. We have revised the discussion as follows.

(Page 32, Line 14-16 – Page 33, Line 1)

Although we have not clearly detected RAGE protein in RAW264.7 cells compared to lung tissue as positive control, AGE-3-induced the suppression of response to LPS was attenuated by FPS-ZM1 and neutralising antibody against RAGE. The RAGE expression in RAW264.7 cells may therefore be quite low.

(Page 37, Line14-16 – Page 38, Line 1-7)

The second limitation is that the evidence for involvement of RAGE on AGE-induced suppression of response to LPS have not adequately established. In western blot analysis for protein expression of RAGE, indistinct bands were observed in lysate of RAW264.7 cells in contrast to lung tissue. In the present study, we did not confirm the involvement of RAGE using knockout or knockdown experiments. High concentration of FPS-ZM1 at 10 μM has inhibitory effect on androgen receptor, benzodiazepine receptor, kappa opioid receptor and melatonin receptor [55]. However, none of these receptors bind to AGEs. In addition, our results showed that FPS-ZM1 alone did not affect LPS-induced CXCL10 release. Although our results remain limitation, we considered that FPS-ZM1 and anti-RAGE antibody inhibit AGEs though blockage of RAGE in the main.

Reference

[55] Drake LR, Brooks AF, Stauff J, Sherman PS, Arteaga J, Koeppe RA, et al. Strategies for PET imaging of the receptor for advanced glycation endproducts (RAGE). J Pharm Anal. 2020;10: 452–465. doi:10.1016/J.JPHA.2020.07.009

***2. Missing from the discussion/model is any suggestion of mechanism. The data in Figure 2 are intriguing. CD14 levels return to normal by 4 h but it takes 24 hr to get significant release of CXCL10. Why is LPS necessary for >16 h when CD14 is restored much earlier? LPS must be doing something more than just reducing CD14 levels. Are there any suggestions on what that might be? How do the authors envision that AGE-3/RAGE engagement reduces CD14 expression and CXCL10 production? And how might AGE/RAGE be impacting the normal LPS signaling for CXCL10?***

**Reply to the comment 2:**

We have revised the discussion as follows (Page 34, Line12-16 – Page 35, Line 1-7).

In general, LPS activates TRIF-dependent signalling and facilitates the transcription of CXCL10 mediated by the phosphorylation of IRF3. Previous report has demonstrated that upregulation of CXCL10 mRNA by LPS began to increase and reached maximal level at 4 h in peritoneal macrophage prepared from mouse [27]. And then CXCL10 protein are produced by translation and released into culture medium. Therefore, we suggest that the time lag is occurred between CD14-initiated LPS uptake and CXCL10 production. Additionally, LPS activate release other proinflammatory cytokines such as TNF-α in macrophage. The TNF-α release from macrophage cells occur within a short period (<4hr) after exposure to LPS [46,47]. TNF-α also increases CXCL10 production in macrophage [48]. It raises possibility that TNF-α released from RAW264.7 cells treated with LPS synergically induce CXCL10 release after long-term exposure to LPS.

(Page 35, Line9-16 – Page 36, Line 1-8)

In the present study, our results suggest that AGEs-induce downregulation of CD14 expression is partially involved in reduction of CXCL10 release in RAW264.7 cells treated with LPS. LPS decreases CD14 expression on cell surface, since LPS binding to CD14 is endocytosed into cell. Although uptake of AGEs is occurred in RAW264.7 cells, anti-CD14 antibody did not affect AGEs uptake, indicating that CD14 independent mechanisms contribute to AGEs uptake. Our previous report has demonstrated that SR-A is associated with AGEs uptake in RAW264.7 cells [15]. It has been reported that stimulation of monocyte by activating agents such as LPS and IFN-γ results in downregulation of CD14 expression on cell surface mediated by shedding of CD14 [49]. CD14 is anchored to the cell membrane by glycosylphosphatidylinositol-anchor [50]. Protease including matrix metalloproteinases (MMPs) contribute to the proteolytic shedding of CD14 [51]. AGE/RAGE signaling increases expression and activity of MMP-9 in macrophage [52]. Therefore, one possibility is that AGE/RAGE decreases CD14 expression on cell surface through excessive shedding of CD14, resulting in decreased CXCL10 production after exposure to LPS.

Reference

[46] Thorley AJ, Ford PA, Giembycz MA, Goldstraw P, Young A, Tetley TD. Differential Regulation of Cytokine Release and Leukocyte Migration by Lipopolysaccharide-Stimulated Primary Human Lung Alveolar Type II Epithelial Cells and Macrophages. J Immunol. 2007;178: 463–473. doi:10.4049/JIMMUNOL.178.1.463

[47] TA H, DM T, SK D, AK C, JW P, A B. Post-transcriptional effects of extracellular pH on tumour necrosis factor-alpha production in RAW 246.7 and J774 A.1 cells. Clin Sci (Lond). 2001;100: 259–266. Available: https://pubmed.ncbi.nlm.nih.gov/11222111/

[48] Qi X-F, Kim D-H, Yoon Y-S, Jin D, Huang X-Z, Li J-H, et al. Essential involvement of cross-talk between IFN-γ and TNF-α in CXCL10 production in human THP-1 monocytes. J Cell Physiol. 2009;220: 690–697. doi:10.1002/jcp.21815

[49] Bazil V, Strominger JL. Shedding as a mechanism of down-modulation of CD14 on stimulated human monocytes. J Immunol. 1991;147: 1567–1574. Available: https://www.jimmunol.org/content/147/5/1567

[50] Haziot A, Chen S, Ferrero E, Low MG, Silber R, Goyert SM. The monocyte differentiation antigen, CD14, is anchored to the cell membrane by a phosphatidylinositol linkage. J Immunol. 1988;141: 547–552. Available: https://www.jimmunol.org/content/141/2/547.short

[51] Senft AP, Korfhagen TR, Whitsett JA, Shapiro SD, LeVine AM. Surfactant Protein-D Regulates Soluble CD14 through Matrix Metalloproteinase-12. J Immunol. 2005;174: 4953–4959. doi:10.4049/JIMMUNOL.174.8.4953

[52] Zhang F, Banker G, Liu X, Suwanabol PA, Lengfeld J, Yamanouchi D, et al. The Novel Function of Advanced Glycation End Products in Regulation of MMP-9 Production. J Surg Res. 2011;171: 871–876. doi:10.1016/J.JSS.2010.04.027

**Minor comments**

***3. No need to define MFI in every figure. In methods and Fig 1 is fine. Same with "n= means the number of exp."***

**Reply to the comment 3:**

As you pointed out, MFI and number of samples define in methods and Fig.1.

***4. Page 22 line 11-12: “not indispensable for LPS uptake”. This means that TLR4 is REQUIRED for endocytosis. I think the authors mean “is not required for LPS uptake” Please replace the phrase “not indispensable” with a clearer description. That is, is TLR4 required for endocytosis? If so, say that. If it is not, then state that clearly that it is not necessary.***

**Reply to the comment 4:**

Thank you for valuable comment. TLR4 is not required for LPS uptake. We have revised the phrase (Page 22, Line 9-11).

CD14 is an essential protein for LPS recognition and uptake. In contrast to CD14, TLR4 contributes to the activation of signalling responses to LPS, although it is not required for LPS uptake [24].

***5. Page 23 line 7 confirmed, not conformed***

**Reply to the comment 5:**

As you pointed out, we have revised the phrase.

***6. Page 23, line 8, but, not bot***

**Reply to the comment 6:**

As you pointed out, we have revised the phrase.

***7. Page 26-27 lines 16 and 1-2 “Furthermore, low dose of AGE-3 inhibited LPS uptake without reduction of CD14 expression in the absence and presence of LPS, indicating that AGE-3 suppresses LPS uptake and CXCL10 production mediated by both CD14 dependent and independent mechanisms.” From Fig 3(B), 20 µg/mL AGE3 significantly inhibits LPS uptake AND there is a trend (not statistically significant but this is only an n=3, Fig 3E) towards reduced CD14. In Fig 3F, the MFIs are so low (and flow is read on a log scale), the differences seen (or not seen) by flow could be a limitation of the sensitivity of the flow machine. In this reviewer’s opinion, the author’s conclusion is not supported by the data and should be removed from the final version. It is noted that the n on Fig 3F is 5 vs 3 for most of the other data. Was this done more times to see if the results were significant?***

**Reply to the comment 7:**

Unlike inhibitory effect of anti-CD14 antibody, AGE-3 slightly decreases LPS uptake and CD14 expression in the presence of LPS. Especially, the effect of low-dose AGE-3 on CD14 expression is controversial. As you pointed out, our results relating to CD14 expression are only obtained by flow cytometry. Other experimental techniques such as immunocytochemistry or western blot for membrane protein are seem necessary to evaluate the effect of low-dose AGE-3 on CD14 expression. We have deleted the conclusion relating to the effect of low-dose AGE-3.

***8. Page 29, lines 9-11: The fact that anti-SR-A Ab suppresses AGE-3 uptake does not confirm that the same concentration of a different (anti-RAGE) Ab would have the same effect. Granted they are both polyclonal goat IgGs but they are not the same Ab and getting blocking with one cannot be generalized to all Abs. This is an overinterpretation of the data.***

**Reply to the comment 8:**

In Page 29, lines 9-11, we would like to have described that SR-A do not contribute to the AGE-3-induced suppression of LPS uptake and CD14 expression. Figs 4A and 4C show that anti-SR-A antibody at 20 µg/mL failed to inhibit AGE-3-induced suppression of LPS uptake and CD14 expression. On the other hand, anti-SR-A antibody at 20 µg/mL suppressed AGE-3 uptake. Therefore, our findings suggest that dose of anti-SR-A antibody at 20 µg/mL are sufficient to suppress effects mediated by AGE-3. In our previous study, we have demonstrated that anti-RAGE antibody at 20 µg/mL failed to inhibit AGE-3 uptake in RAW264.7 cells. We have revised the sentence as follows (Page 28, Line 16 – Page 29, Line 1-3).

However, anti-SR-A antibody suppressed the uptake of AGE-3 in RAW264.7 cells (S5 Fig) indicating that the concentration of anti-SR-A antibody sufficient to suppress effects mediated by AGE-3. Therefore, SR-A do not contribute to the AGE-3-induced suppression of LPS uptake and CD14 expression.

***9. Page 29: “We verified the protein expression of RAGE in RAW264.7 cells by using western blot. Consistent with previous study [33], the two bands, which represent pre and post glycation type of RAGE protein, in the vicinity of 50 kDa were observed in mouse lung tissue used as positive control (S6 Fig). The protein sample prepared from RAW264.7 cells showed weak band in the vicinity of 50 kDa, indicating that RAGE is a functional receptor for AGEs despite expression are low in RAW264.7 cells.” The very light band at ~50K in RAW cells does not convincingly show that RAGE is expressed in this cell line. The gel seems overexposed as indicated by the very dark RAGE bands in the positive control. A lower exposure would show no bands in the 50 KDa range. Even so, assuming this clone of RAW cells is the same as that reported in ref 33, a PCR showing message expression would be more convincing than the Western shown. Additionally, the fact that there may be RAGE expression in these cells (questionable as this is not a clean gel and there are many bands of equal or higher intensity to the putative RAGE band), the presence of protein does not demonstrate that RAGE is a FUNCTIONAL receptor.***

**Reply to the comment 9:**

RAGE is abundantly expressed in lung tissue compared to other tissue. From database of gene expression profile (BioGPS: http://biogps.org), RAGE expression in lung tissue is almost two thousand-fold higher than other tissue or cells including RAW264.7 cells. Our results obtained from western blot show RAGE bands has little detected in RAW264.7 cells under lower exposure condition. Although source of RAW264.7 cells using ref 33 is unclear, protein expression is important for evaluate receptor function compared to mRNA expression. In the present study, we found that both FPS-ZM1 and anti-RAGE antibody attenuate AGE-3-induced suppression of LPS uptake, CD14 expression and CXCL10 release form RAW264.7 cells treated with LPS. However, we did not confirm the involvement of RAGE using knockout or knockdown experiments. The limitation of the present study is lack of evidence for involvement of RAGE on the AGE-induced suppression of LPS response inRAW264.7 cells. Although both FPS-ZM1 and anti-RAGE antibody attenuate the effect of AGE-3, protein expression of RAGE in RAW264.7 cells is unclear. We did not confirm the involvement of RAGE using knockout or knockdown experiments. We have added the discussion ((Page 38, Line16 – Page 39, Line 1-9). Please see point “Reply to the comment 1” above.

***10. Page 36: What do the authors mean that “distribution of cell population was confused”?***

**Reply to the comment 10:**

In flow cytometric analysis using cell line, distribution of cell population based on cell size and intracellular complexity converge one population, because cells are homogeneous and has uniform size. LPS treatment results in confusion of cell distribution. It is possibility that the LPS treatment or the experimental techniques, which prepare cell sample for flow cytometric analysis, potentially damage cell. We considered that confusion of cell distribution induced by LPS treatment mean cell damage. We have added the text (Page 37, Line 6-7).

***11. Finally, the English still needs to corrected. Some of the edits to "correct" English are wrong. Please have native English speaker (or editor) correct so that verb and subject match.***

**Reply to the comment 11:**

We have carefully corrected our manuscript.
